# Slipping and tripping: fall injuries in adults associated with rugs and carpets

**DOI:** 10.5249/jivr.v5i1.177

**Published:** 2013-01

**Authors:** Tony Rosen, Karin A. Mack, Rita K. Noonan

**Affiliations:** ^*a*^National Center for Injury Prevention & Control, Division of Unintentional Injury Prevention, Centers for Disease Control and Prevention, Atlanta, GA USA.

**Keywords:** Floors, Floor coverings, Fall, Injury

## Abstract

**Background:**

Falls are a leading cause of unintentional injury among adults age 65 years and older. Loose, unsecured rugs and damaged carpets with curled edges, are recognized environmental hazards that may contribute to falls. To characterize nonfatal, unintentional fall-related injuries associated with rugs and carpets in adults aged 65 years and older.

**Methods:**

We conducted a retrospective analysis of surveillance data of injuries treated in hospital emergency departments (EDs) during 2001–2008. We used the National Electronic Injury Surveillance System-All Injury Program, which collects data from a nationally representative stratified probability sample of 66 U.S. hospital EDs. Sample weights were used to make national estimates.

**Results:**

Annually, an estimated 37,991 adults age 65 years or older were treated in U.S. EDs for falls associated with carpets (54.2%) and rugs (45.8%). Most falls (72.8%) occurred at home. Women represented 80.2% of fall injuries. The most common location for fall injuries in the home was the bathroom (35.7%). Frequent fall injuries occurred at the transition between carpet/rug and non-carpet/rug, on wet carpets or rugs, and while hurrying to the bathroom.

**Conclusions:**

Fall injuries associated with rugs and carpets are common and may cause potentially severe injuries. Older adults, their caregivers, and emergency and primary care physicians should be aware of the significant risk for fall injuries and of environmental modifications that may reduce that risk.

## Introduction

Falls are the leading cause of injuries requiring emergency treatment in adults aged 65 and older and lead to more hospital admission and deaths than any other type of trauma.^1^ They are also associated with increased premature mortality,^2,3^ loss of independence,^4,5^ and nursing home placement.^6^ In addition, fear of falling may lead to avoidance of activities,^7-10^ potentially reducing physical fitness and mobility and increaseing social isolation, time spent at home, and depression.

Falls in the elderly rarely have a single isolated cause but typically occur because of the interaction of multiple contributing factors. Researchers often classify these as intrinsic and extrinsic factors.^11,12^ Intrinsic factors include individual susceptibilities that increase fall risk such as chronic functional impairments (e.g. reduced balance or vision loss) and acute illnesses (e.g. pneumonia or myocardial infarction).^11-17^ Extrinsic or environmental risk factors, which include unsafe walking surfaces, obstacles in path, inappropriate footwear, and poor lighting,^11-17^ have been shown to play a part in approximately half of all home falls,^18^ and their importance in increasing risk has long been recognized by researchers^19,20^ and occupational therapists working with older adults.^21,22^

Loose throw rugs and area carpets with curled edges or folds are among the extrinsic factors most frequently mentioned in the literature as unsafe and potentially increasing fall risk.^20,23,24^ Research has shown that hazardous rugs and carpets may be the most common environmental hazard in the homes of older adults,^25^ with one study finding loose throw rugs in nearly 78% of the homes,^26^ curled carpet edges in more than 35%,^26^ an average of more than 11 rugs without nonslip backing in each home.^25^ These hazards are even more common in homes of frail older adults with disabilities,^27^ who are at higher risk for falls. Evidence also exists that these flooring types may increase risk of serious fall-related injury. Case control studies have found that both floor mats in hallways and bathmats significantly increased risk of hip fractures^21^ and that loose rugs / mats and flooring were among the most common objects in the home associated with falls resulting in hip fractures.^28^

Despite the intuitive connection between environmental hazards such as loose rugs and curled carpet edges and increased risk of falls, longitudinal research has shown mixed results. A hospital-based randomized controlled trial found that more falls occurred in the group housed in rooms with carpeted flooring than in rooms with vinyl flooring.^29^ Other studies, however, have not shown an association,^30^ and in one, the presence of loose throw rugs was actually associated with a decreased risk of fall among adults 65–84 years old.^31^

We chose to examine falls involving these flooring types, as the size and scope of this public health problem has not yet been well-defined. Our study objective is to more fully quantify and characterize fall injuries associated with rugs and carpets in older adults. To do this, we provide the first published U.S. national estimates of non-fatal fall-related injuries associated with these flooring types among adults aged 65 years and older that required emergency care. We hope to use this information to understand the public health burden of these injuries and to identify and prioritize appropriate intervention strategies.

## Methods

**Study Design and Data Source**

This study analyzed data from the 2001–2008 National Electronic Injury Surveillance System-All Injury Program (NEISS-AIP), a collaborative effort between the Centers for Disease Control and Prevention’s National Center for Injury Prevention and Control and the U.S. Consumer Product Safety Commission. NEISS-AIP collects data for emergency department visits for all types and causes of injuries from a nationally representative stratified probability sample of 66 hospitals in the United States and its territories having at least six beds and providing 24-hour emergency services. For each initial ED visit, coders record characteristics, including age, sex, and disposition of patients. Also recorded is one principal diagnosis, usually the most severe, as determined by the physician or healthcare provider and recorded in the medical chart, and one primary part of the body injured, on the basis of a fixed number of categories. International Classification of Diseases, Ninth Revision, Clinical Modification (ICD-9-CM) diagnosis codes are not available in the medical record at the time these data are collected; therefore, specific types of injuries, such as hip fractures, cannot be accurately identified. Brief two-line narratives about the circumstances of the injury are recorded for each case. Also, up to two products that are involved in an injury incident may be coded, with “product” broadly defined to include consumer products (e.g., walkers, canes, shoes) and other objects that are involved (e.g., floors, walls, or stairs). NEISS-AIP defines a fall injury as one received when a person descends because of the force of gravity and strikes a surface at the same or lower level. More information about NEISS-AIP is available at: http://www.cdc.gov/ncipc/wisqars /nonfatal/datasources.htm.

**Study Sample**

For our analysis, cases were defined as adults aged 65 years and older treated at an NEISS-AIP ED for a non-fatal, unintentional fall injury that occurred between January 1, 2001 and December 31, 2008 and that involved a rug or carpet. All cases with product codes 0612 (runners, throw rugs, or door mats, excluding bathmats), 0613 (room-sized, wall-to-wall, or outdoor carpeting, excluding runners), and/or 0676 (rugs or carpets, not specified) were included in this analysis. Additional cases were identified if the brief narrative contained mention of “rug” or “carpet”. Falls involving mats were excluded from analyses because the number of cases was too small (n=146) for meaningful analysis.

**Qualitative Case Finding and Data Analysis**

The narratives of all potential cases were individually reviewed and excluded if (1) the fall did not actually include a rug or carpet, (2) there was a clear alternative mechanism of fall described (e.g., patient tripped over the leg of a table and landed on carpet), (3) the subject was not standing on the floor or walking when the fall occurred (e.g., patient rolled out of bed and fell onto carpet or patient was standing on a chair, fell, and landed on rug), (4) if the carpet or rug was on a stair, or (5) it was not clear whether the fall involved a rug/carpet (e.g., the patient fell down several steps and landed on the carpeted floor). We excluded stair falls because the circumstances of falls on stairs are sufficiently different from falls on a flat surface to warrant separate analysis. Risk of falling on stairs depends on several factors in addition to floor covering, including, for example, the number of stairs, whether the person is ascending or descending, whether the person is at the top, middle, or bottom of the stairs, and whether guard rails are present. 

Occupational injuries were identified by use of the work-related/occupational code and were excluded from analyses since work-related injuries would be covered by occupational safety standards. Despite these exclusion, the final number of useable cases was substantial (n=4,015).

Because rugs and carpets were included together in the same NEISS-AIP product categories, we used the text in the brief narrative comment to categorize them. In addition, because detailed information about the location of injury, such as room within the home or specific location outside the home (e.g., store, professional office, place of worship) is not explicitly captured in the NEISS-AIP dataset, the authors individually reviewed the brief narratives for each case and recorded this information when it was available. For example, bathroom was recorded if the narrative mentioned explicitly that the injury occurred in the bathroom or if the injury occurred when a patient tripped on a rug while getting out of the bathtub. For falls in the home, room could be specifically coded in 12% of the cases, while 88% remained unknown. For falls outside the home, location could be specifically coded in 87% of the cases, while 13% remained unknown. 

**Quantitative Data Analysis**

All estimates were based on weighted data for 4,015 ED visits. Ninety-five percent confidence intervals (CI) were calculated by use of a direct variance estimation procedure that accounted for the sample weights and the complex sampling design. Estimates with coefficients of variation (CVs) greater than 30% were considered unstable, and rates and confidence intervals in such instances were not reported. Analyses were conducted by use of SPSS (SPSS Inc., Chicago, IL) complex samples to account for the sampling design. 

## Results

**Quantitative Findings**

On the basis of 4,015 cases in this sample, an estimated 37,991 fall injuries associated with rugs or carpets in adults aged 65 years and older were treated annually in U.S. EDs (Table 1). Approximately 54.2% of the fall injuries were associated with carpets and 45.8% with rugs. A large majority of fall injuries occurred at home (72.8%), while 15.2% occurred at locations outside the home and 12.0% did not include enough information to allow determination of the location. The age group accounting for the most injuries was 75–84 (45.2%). The body part most commonly injured was the head/neck (27.9%). Most patients (72.7%) were treated and then released after being seen in the emergency department. 

**Table:** Table 1: **Annual estimates of nonfatal unintentional fall injuries in people age 65 years and older treated in US emergency departments and associated with carpets and rugs, 2001–2008.**

Characteristic	Total of Carpets and Rugs			Carpets			Rugs		
	Annual estimate	Percent	95% CI	Annual estimate	Percent	95% CI	Annual estimate	Percent	95% CI
Total	37991	100.0		20583	54.2	47.9-60.3	17408	45.8	39.7-52.1
Gender									
Men	7512	19.8	18.3-21.3	3784	18.4	16.6-20.3	3728	21.4	19.1-23.9
Women	30467	80.2	78.7-81.7	16787	81.6	79.7-83.4	13680	78.6	76.1-80.9
Location									
Home	27649	72.8	67.6-77.4	14825	72.0	65.6-77.7	12823	73.7	68.3-78.4
Outside home	5785	15.2	12.6-18.2	3306	16.1	12.7-20.1	2479	14.2	11.8-17.0
Unknown	4557	12.0	7.8-18.0	2452	11.9	7.7-18.0	2105	12.1	7.6-18.8
Age group (years)									
65-74	8950	23.6	21.3-25.9	4254	20.7	18.2-23.4	4696	27.0	24.1-30.1
75-84	16159	42.5	40.0-45.1	8677	42.2	38.5-46.0	7483	43.0	40.5-45.5
85+	12882	33.9	31.5-36.4	7653	37.2	34.0-40.5	5229	30.3	27.5-32.7
Injury Diagnosis									
Contusion	10016	26.0	23.7-29.2	5640	27.4	24.6-30.4	4376	25.1	21.7-28.9
Fracture	15861	41.7	39.2-44.4	8775	42.6	40.0-45.3	7086	40.7	36.8-44.7
Laceration	4624	12.2	10.5-14.0	1988	9.7	8.0-11.6	2636	15.1	12.9-17.7
Internal Injury	2551	6.7	5.2-8.6	1467	7.1	5.6-9.0	1084	6.2	4.4-8.7
Strain/sprain	2859	7.5	6.5-8.7	1776	8.6	7.2-10.2	1083	6.2	4.9-7.9
Other	2080	5.5	4.3-6.9	937	4.6	3.5-6.0	1143	6.6	5.0-8.6
Body part injured									
Head/neck	10584	27.9	25.8-30.0	5388	26.2	23.5-29.0	5197	29.9	26.8-33.1
Upper trunk	5333	14.0	12.9-15.3	2817	13.7	12.0-15.6	2516	14.5	12.6-16.5
Lower trunk	9205	24.2	22.5-26.1	5574	27.1	25.2-29.0	3630	20.9	18.2-23.8
Arm/hand	5926	18.2	17.1-19.4	3705	18.0	16.5-19.6	3222	18.5	16.3-20.9
Leg/Foot	5726	15.1	13.9-16.3	2988	14.5	13.1-16.1	2738	15.7	13.9-17.8
Other/unknown	217	0.6		112	0.5		104	0.6	
Disposition									
Treated & released	27634	72.7	69.4-75.8	14689	71.4	68.0-74.5	12945	74.4	69.7-78.5
Hospitalized or transferred	9988	26.3	23.3-29.5	5726	27.8	24.7-31.2	4262	24.5	20.5-28.9
Other	370	0.1		168	0.8		201	1.2	

Table 1 also compares characteristics of fall injuries associated with carpets and rugs. Generally, we found few significant differences in injuries associated with the two flooring types. Notably, a significantly larger percentage of the younger old (65-74 years) were injured on rugs rather than carpets (27.0% vs. 20.7%), while a significantly larger percentage of the older old (85+ years) were injured on carpets (37.2% vs. 30.3%). Also, a significantly larger percentage of patients falling on rugs suffered laceration than those falling on carpets (15.1% vs. 9.7%), while a significantly larger percentage of carpet fallers injured their lower trunk (27.1% vs. 20.9%).

Women represented 80.2% of these rug and carpet-associated fall-related injuries. Characteristics of the injuries for men and women were generally similar, including similar rates of hospitalization, with a few notable exceptions. Women had significantly higher percentages of fracture (43.5% vs. 34.5%) (Table 2). Men had significantly higher percentages of head/neck injuries (33.7% vs. 26.4%).

**Table:** Table 2: **Annual estimates by gender of nonfatal unintentional fall injuries in people age 65 years and older treated in US emergency departments and associated with carpets and rugs, 2001–2008.**

Characteristic	Men			Women		
	Annual estimate	Percent	95% CI	Annual estimate	Percent	95% CI
Flooring Type						
Carpet	3784	50.4	43.5-57.2	16787	55.1	48.7-61.4
Rug	3728	49.6	42.8-56.5	13680	44.9	38.6-51.3
Location						
Home	5485	73.0	66.5-78.7	22150	72.7	67.5-77.4
Outside home	1054	14.0	10.9-18.0	4731	15.5	12.7-18.8
Unknown	972	12.9	7.9-20.1	3585	11.8	7.7-17.6
Age group (years)						
65-74	1940	25.8	21.8-30.3	7010	23.0	20.7-25.5
75-84	3167	42.2	38.6-45.8	12979	42.6	40.0-45.2
85+	2405	32.0	27.6-36.8	10477	34.4	31.8-37.1
Injury Diagnosis						
Contusion	2084	27.7	23.3-32.7	7932	26.0	23.4-28.8
Fracture	2592	34.5	29.2-40.2	13256	43.5	40.9-46.1
Laceration	1142	15.2	11.8-19.4	3482	11.4	9.7-13.4
Internal Injury	692	9.2	6.9-12.1	1859	6.1	4.7-7.9
Strain/sprain	551	7.3	5.2-10.3	2309	7.6	6.5-8.9
Other	452	6.0	4.2-8.4	1629	5.3	4.2-6.8
Body part injured						
Head/neck	2528	33.7	29.9-37.7	8056	26.4	24.4-28.6
Upper trunk	1170	15.6	12.8-18.8	4163	13.7	12.2-15.2
Lower trunk	1652	22.0	18.6-25.8	7540	24.7	22.8-26.8
Arm/hand	1123	14.9	12.0-18.5	5804	19.0	17.9-20.3
Leg/Foot	1012	13.5	11.7-15.5	4714	15.5	14.1-17.0
Other/unknown	28	0.4	--	189	0.6	--
Disposition						
Treated & released	5537	73.7	67.5-79.1	22084	72.5	69.2-75.6
Hospitalized or transferred	1833	24.4	19.2-30.4	8155	26.8	23.7-30.0
Other	141	1.9	--	228	0.7	--

**Figure 1: Room/location in home where fall injuries occurred (n=333):**
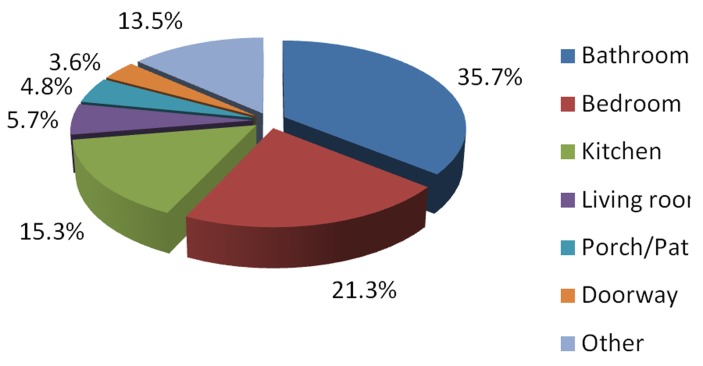


We evaluated the location in the home where fall injuries associated with rugs or carpets occurred for case files for which this information was available (333 of 4,015 cases; Figure 1). We excluded injuries occurring on carpeted or rug-covered stairs. The most common locations in the home where these injuries occurred were the bathroom (35.7%) and bedroom (21.3%). For falls occurring outside the home (detail not shown), the most common locations for fall injuries were nursing home / assisted living / group home (25.8%) and store / shopping mall / bank (19.2%).

**Qualitative Findings**

Through qualitative review of the case narratives, we noted characteristic circumstances surrounding fall injuries. Older adults commonly fell and injured themselves at the transition between carpet/rug and non-carpet/rug. Wet carpets or rugs often contributed to falls. Patients frequently suffered an injurious fall while “hurrying to” or “trying to get to” the bathroom.

## Discussion

This study is, to our knowledge, the first national report of non-fatal, unintentional fall injuries associated with a rug or carpet and sustained by adults age 65 years or older who were treated in EDs. With nearly 38,000 adults requiring emergency treatment annually, rug and carpet-associated fall injuries are clearly an issue worthy of public health attention, although in the context of over 2.2 million fall injuries annually in older adults requiring emergency treatment,^1^ this is just one piece of a much larger picture.

Several aspects of rug and carpet-associated fall injuries in our study are consistent with fall injuries in general among older adults, suggesting that the injuries suffered may be similar to those from falls not associated with these flooring types. Most of the injuries occurred in the home. This is consistent with falls research, which has suggested that 70–80% of falls occur in and around an older adult’s home.^5,32^ This is likely because community-dwelling older adults, particularly those who are frail and therefore at increased fall risk, spend most of their time at home and, therefore, have more exposure to environmental hazards in the home. Our results showed generally increasing percentages of fall injuries in advancing age groups, with a decrease in the highest age group. This is consistent with previous research, which has shown that risk of experiencing a fall at home increases with advancing age.^33-35^

In our study, women represented more than 80% of those injured. Evidence exists that older women may fall more frequently than older men, perhaps because of greater impairment of balance and the muscle power required to counteract destabilization.^36-41^ Women are also more likely to suffer an injury from falling.^42^ Notably, that study found fractures were 2.2 times more common in women than in men and injuries of the arm/hand were 2.0 times as common. This increased risk of fractures in women, which is consistent with our findings, is thought to be due to higher rates of osteoporosis and decreased bone mass.^43^ In addition, cultural gender roles may have an impact on these findings, as women may be more likely than men to seeking medical care sooner after the injury.^44^

In our study, a significantly higher percentage of male fallers suffered head and neck injuries. Previous research has shown that adult men are more likely than women to suffer a traumatic brain injury^45,46^ and were more likely to require hospitalization.^47^ This may be due to greater vulnerability or other differences in the underlying causes or circumstances of their falls.^42^ One notable difference between our findings and current falls research in general is that we found similar hospitalization rates for men and women for rug and carpet-related fall injuries, despite recent research on fall injuries in general, which found that women are 1.8 times more likely to be hospitalized.^42^ Our finding of rough equivalence may be potentially explained by an increase in hospitalization for fracture-related injuries for women in our sample being balanced by greater hospitalization rates for head / neck injury for men, or it may provide evidence that rug and carpet--associated falls are different from other falls in another way.

That older adults commonly suffered fall injuries at the edge between carpet and non-carpeted floor in this study is not surprising. Transition areas often have differences in floor height^48^ and may pose a problem for the rubber tips of canes or crutches.^49^ Transitions frequently occur at areas of light differences, which pose additional risks.^50-52^ In this sample, the bathroom was the most frequent location in the home for fall injuries to occur, supporting recent literature that suggests that bathroom falls are common.^53^ Previous reports have found that bathrooms are the most common site for environmental hazards in the home,^54^ with 45% of older adult fallers having throw rugs in their bathrooms.^31^ These rugs are likely to get wet, making them potentially even more hazardous, particularly if they are not secured to the floor. Older adults engage in balance-displacing activities in the bathroom, such as transferring to/from the bathtub and toilet.

Our work is subject to several limitations. We cannot evaluate how many steps those in the sample took on rugs and carpets vs. other flooring types, so it is not possible to use analytic epidemiology to evaluate risk. In addition, this report includes only non-fatal injuries treated in EDs. We are missing injuries that were treated in physician offices, free-standing medical centers, or other clinics, as well as those injuries that did not require medical attention. Injuries that proved fatal before or in the ED were excluded because NEISS-AIP does not provide detailed information about fatal injuries. However, only approximately 0.5% of unintentional injuries result in death.^1^

Also, we relied on the product code and the two-line brief narrative comment in NEISS-AIP to categorize these fall injuries and to decide which cases to exclude. These brief narratives are variable in their clarity and comprehensiveness and they rely on accurate and complete patient history, physician documentation in the medical record, and abstraction for the NEISS database. One strength of our approach, however, was that by qualitatively evaluating the data, we were able to glean details that may be important for fall prevention programs including an emphasis on the transition between carpet/rug and non-carpet/rug, wet carpet or rugs, and situations such as “hurrying to the bathroom.”

Further, as most of the fall injuries under study were likely unwitnessed, the causative relevance of the rug or carpet as an environmental hazard depends on the victim’s self-report, including the victim’s identification of the hazard and willingness to report it. This self-reporting method may have led to over- and under-reporting, but given the tendency for older persons and their family members to blame the environment for falls, over-reporting is more probable.^32^

In addition, identifying falls that are meaningfully associated with a flooring type is challenging because nearly all fall victims ultimately land on the floor, whether or not it played an important causal role in the event. We have tried to systematically remove from our analysis all files for which the rug or carpet played only an obviously incidental role (a patient who syncopized and landed on the carpet, for example). 

Rugs and carpets may vary dramatically in such characteristics as size, fiber type, pile height, face weight, fiber density, color, pattern, and padding thickness, all of which may significantly affect the associated risk of fall and fall injury and none of which we were able to capture in this study.

Finally, our ability to draw conclusions about in which rooms in the home falls most commonly occurred was limited by the absence of this information in the narrative comments of a large percentage of the cases.

## Conclusion

Falls in the elderly are an important public health problem, and our research demonstrates that a significant number of these falls are associated with rugs and carpets. Older adults and their families and caregivers should be aware of these risks. Emergency physicians, when treating an older patient for a fall injury, may consider asking the patient about the environmental circumstances surrounding the incident and suggesting potential environmental modifications. Primary care physicians should counsel their patients at high risk for falls to be mindful of potentially dangerous rugs or carpets. Fall injuries may be affected by securing rugs with adhesive tape or using non-skid backing, checking for and repairing curled carpet edges. Bathrooms, transitions between rug/carpeted areas and non-rug/carpeted areas, and wet rugs or carpets are particularly dangerous. Notably, evidence to date only shows reduction in falls for home modification programs when implemented by an occupational therapist.^55^ Environmental modifications should be combined with other effective fall prevention strategies such exercise to increase lower body strength, regular vision checks, and frequent assessment for fall risks including a medication review by health care providers.^56^ More research is needed to determine the safest flooring types for older adults and other environmental modifications that may reduce falls. Increasing awareness of the potential hazards associated with rugs and carpets, combined with simple environmental changes may benefit older adults by decreasing the risk for fall injuries.
